# Bilateral follicular thyroid carcinoma with large sternal metastasis: Case report and review of the literature

**DOI:** 10.1016/j.ijscr.2023.108973

**Published:** 2023-10-24

**Authors:** Mohamed S. Al Hassan, Walid El Ansari, Hamza Said Wali, Ehab Massad, Adham Darweesh, Abdelrahman Abdelaal

**Affiliations:** a Department of General Surgery, Hamad General Hospital, Doha, Qatar; b Department of Surgery, Hamad General Hospital, Doha, Qatar; c College of Medicine, Qatar University, Doha, Qatar; d Weill Cornell Medicine – Qatar, Doha, Qatar; e Department of Emergency Medicine, Hamad General Hospital, Doha, Qatar; f Department of Thoracic Surgery, Hamad General Hospital, Doha, Qatar; g Department of Clinical Imaging, Hamad General Hospital, Doha, Qatar

**Keywords:** Follicular thyroid carcinoma, Sternal metastases, Sternectomy, Radioactive iodine therapy, Lenvatinib, Case report

## Abstract

**Introduction and importance:**

Follicular thyroid cancer (FTC) typically spreads hematogenously, with bone metastasis being worrisome, often appearing to be resistant to radioactive iodine (RAI) therapy. Metastasis to sternum is exceedingly rare.

**Case presentation:**

A 43-year-old Egyptian male presented with chest tightness, cough, and shortness of breath. He was initially treated as bronchial asthma. Later, he was referred to our thyroid surgery clinic as a case of goitre and palpable sternal mass. He looked clinically well, with enlarged anterior neck mass and visible sternal mass, no lymphadenopathy. Laboratory tests showed thyroid-stimulating hormone levels within normal (2.13 mIU/L), and mildly decreased FT4 (10.3 pmol/L). Neck/chest CT demonstrated multinodular goitre with retrosternal extension, expansile lytic lesion in the sternum, and bilateral lung metastases. Thyroid fine needle aspiration and cytology showed FLUS, and true cut biopsy from the sternal lesion showed invasive FTC.

**Discussion:**

Rare bilateral FTC presenting as slow-growing sternal metastasis. The patient underwent total thyroidectomy, followed by high dose RAI therapy, and concluded with sternectomy and reconstruction surgery repair using polymethyl methacrylate wrapped in proline mesh. On follow-up, he received further RAI ablation therapy and became RAI refractory. He then received systemic therapy (Lenvatinib). Most recent follow up showed that the disease was controlled (low volume cancer) and he was tolerating treatment well with no reported symptoms.

**Conclusion:**

Bilateral FTC with sternal metastasis is rare, and can be treated with total thyroidectomy, sternectomy and reconstruction, followed by RAI therapy and systemic therapy where required, hence inferring real survival benefit.

## Highlights

• We present bilateral follicular thyroid cancer with retrosternal extension, lytic lesion in sternum, and lung metastases.
• Management included thyroidectomy, high dose radioactive iodine, and concluded with sternectomy and reconstruction surgery.
• Patient became RAI refractory and received systemic therapy (Lenvatinib).
• The disease was controlled (low volume cancer) and he was tolerating well without any reported symptoms.

## 1 Introduction

The most common cancers of the endocrine organs occur in the thyroid gland, with follicular thyroid cancer (FTC) being the second most common thyroid malignancy after papillary thyroid cancer (PTC) [1]. Peak incidence of FTC occurs around a mean age of 60 years [2]. These patients are most commonly asymptomatic, presenting with an enlarged thyroid secondary to a nodule in either or both lobes [2].

FTC typically spreads via hematogenous dissemination, resulting in distant metastases [3]. Despite this, metastasis to lymph nodes is not common (<10 % of cases) [4]. The skeletal system is the second most frequent metastatic destination for differentiated thyroid cancer (DTC) after the lung, involving 2.3–12.7 % of DTC cases [[5], [6], [7]]. Metastases to bone are worrisome in FTC, as they often carry resistance to radioactive iodine (RAI) therapy, rendering their management difficult [8]. FTC is associated with a 10-year survival rate of 80–98 %, dropping to as low as 12 % when bone metastasis is present [9].

We report a 43-year-old Egyptian male with FTC, presenting with an unusual asthma-like exacerbation, later found to have thyroid malignancy with direct sternal invasion. He was managed with multiple stepwise surgeries, radioactive iodine (RAI) therapy, followed by systemic therapy (Lenvatinib). We report this case in line with the updated consensus-based surgical case report (SCARE) guidelines [10].

## 2 Presentation of case

**Fig. 1:**
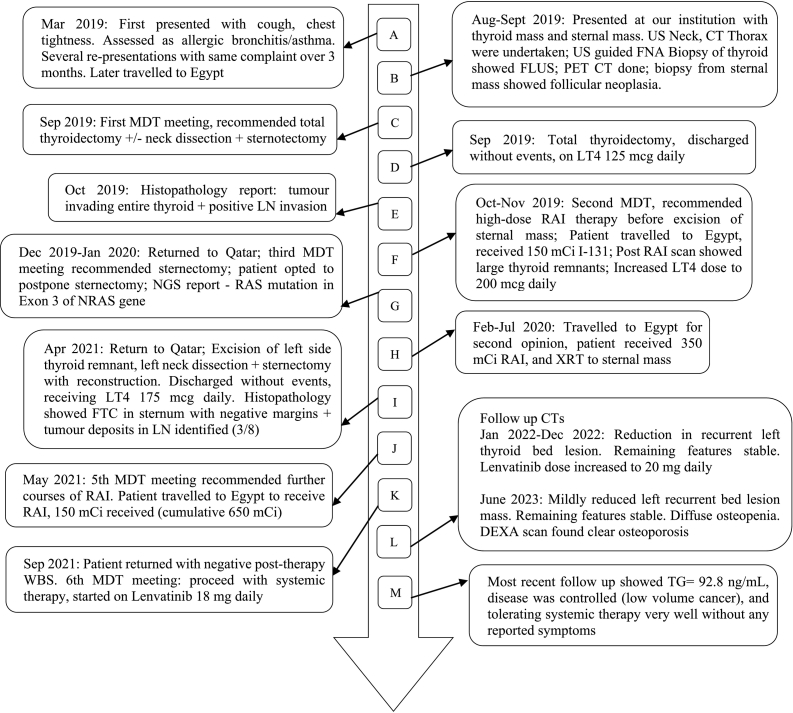
Timeline and sequence of events over 2 years. CT: Computer tomography; DEXA: dual-energy X-ray absorptiometry; FLUS: Follicular Lesion of Undetermined Significance; FNA: Fine-needle Aspiration; FTC: follicular thyroid cancer; LN: Lymph Node; LT4: Levothyroxine; MDT: Multidisciplinary Team; mcg: Micrograms; NGS: Next generation sequencing; NRAS: Neuroblastoma rat sarcoma; PET: Positron Emission Tomography; RAI: Radio-active Iodine; RAS: rat sarcoma; TG: Thyroglobulin; US: Ultrasound; mCi: Millicurie; WBS: Whole body scan; XRT: External beam radiotherapy.

A 43-year-old Egyptian male presented to his local primary healthcare centre complaining of chest tightness, cough, and shortness of breath over a 3-month period. Given his childhood history of asthma, he was treated as a case of bronchial asthma. The patient later travelled to his home country (Egypt); and there he was noted to have a sternal mass and a nodular thyroid gland. Ultrasound (US) and computed tomography (CT) imaging were performed. Fig. 1 depicts the patient's timeline and sequence of events.

**Fig. 2:**
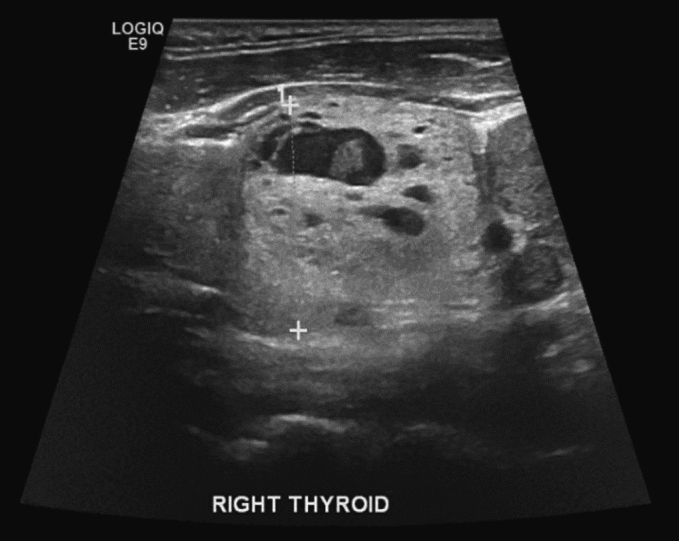
US Thyroid: right lobe (26 mm) with multiple nodules, the largest being complex heterogenous (hyperechoic and cystic) nodule (27 × 23 × 31 mm) with calcifications within, and increased vascularity around the nodule.

**Fig. 3:**
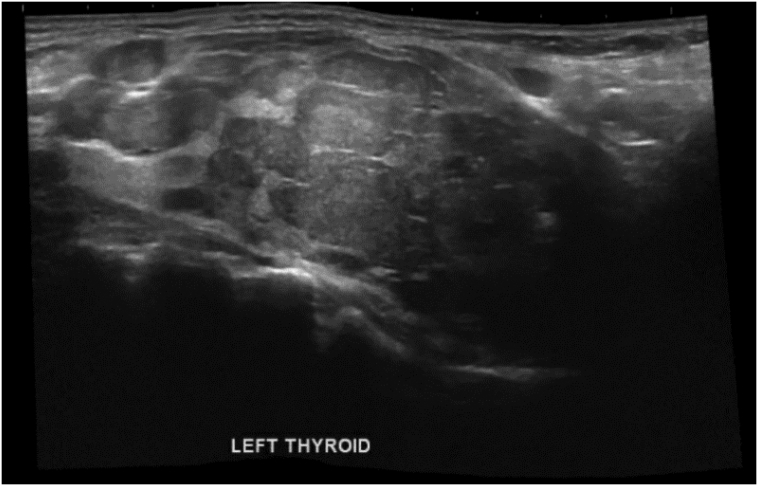
US Thyroid: left lobe (33 mm), with complex heterogenous (hyperechoic and cystic) nodule (37 × 40 × 48 mm), with increased vascularity around and within the nodule.

**Fig. 4:**
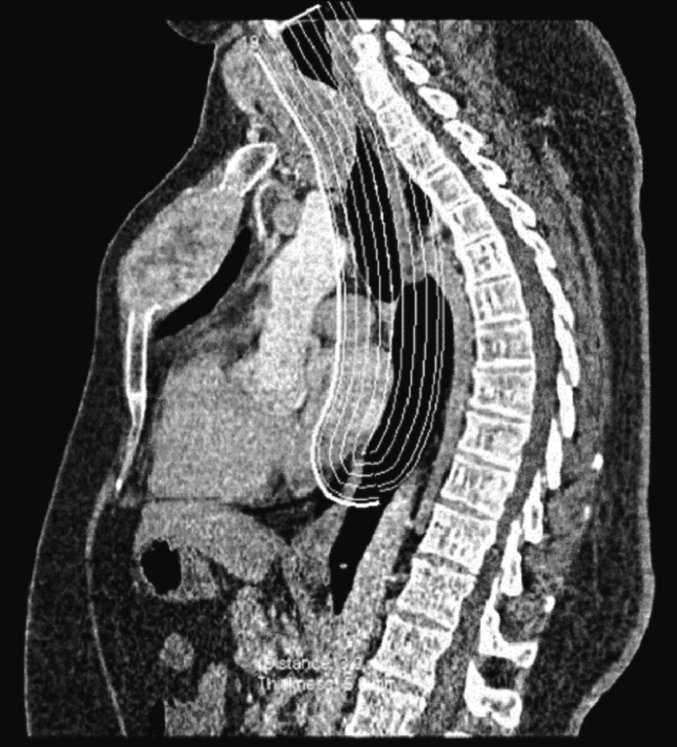
CT scan: showing enlarged nodular thyroid with 2.8 cm retrosternal extension.

**Fig. 5:**
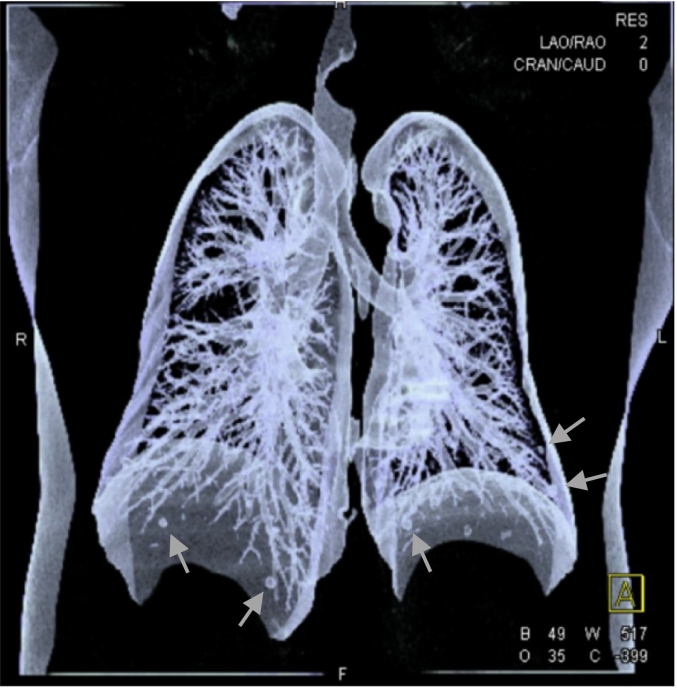
CT scan of the chest: demonstrating several nodules (green arrows). (For interpretation of the references to colour in this figure legend, the reader is referred to the web version of this article.)

The patient presented to us again in Qatar, with imaging reports from Egypt. The imaging from Egypt comprised neck US and chest CT. We repeated the thyroid US, and it redemonstrated the findings from the patient's visit to Egypt. There were multiple nodules in both thyroid lobes, as well as a submandibular lymph node (Fig. 2, Fig. 3). Chest CT demonstrated a multinodular goitre with retrosternal extension about 2.8 cm below the sternum, an expansile lytic lesion in the sternum (5.1 × 4.9 × 9 cm), and bilateral lung metastases (Fig. 4, Fig. 5). Fine needle aspiration and cytology (FNAC) showed features of left thyroid lobe nodule, consistent with follicular lesion of undetermined significance (FLUS). US-guided core needle biopsy of the chest wall mass confirmed abnormal thyroid tissue reminiscent of follicular neoplasia.

**Fig. 6:**
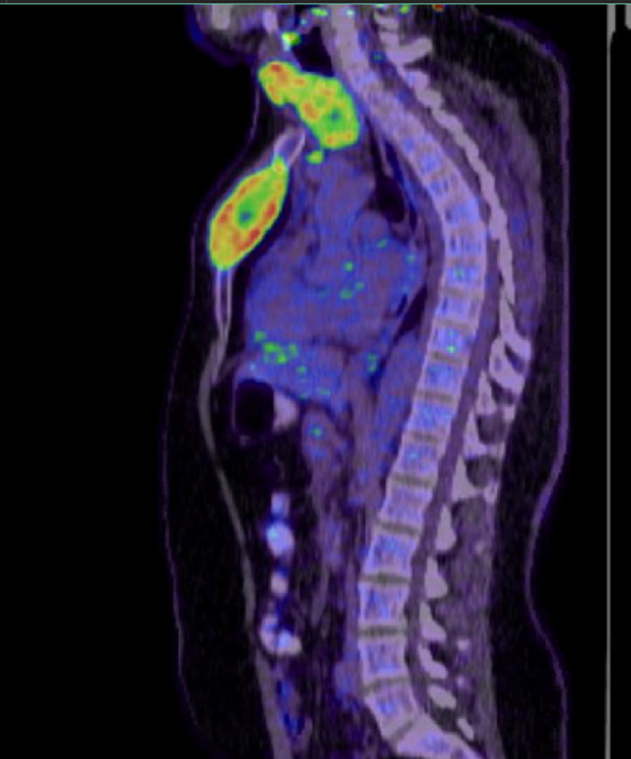
PET-CT scan: showing intensely hypermetabolic expansile lytic lesion in the sternum.

Positron emission tomography and computed tomography (PET-CT) scan (Fig. 6) showed an enlarged multi-nodular thyroid gland compressing the trachea to the right side, an intensely hypermetabolic expansile lytic lesion in the sternum, and bilateral lung nodules with detectable uptake.

The case was discussed at our thyroid multiple disciplinary team (MDT) meeting, and the decision was to undertake a total thyroidectomy due to the patient's respiratory complaints, sternectomy and reconstruction, followed by postoperative high dose RAI ablation therapy. Intra-operatively, the thyroid tumour was compressing but not invading the trachea, with attachments to the strap muscles. Central lymph node sampling was done. The thoracic surgeon decided to reserve the sternectomy for a later date. Pathology report on the excised thyroid was consistent with widely invasive FTC with positive margins and lymph nodes, stage II (T4aN1aM1).

The case was presented at a second MDT meeting (Fig. 1, Section F) for further discussion and planning, and the decision was to administer high dose RAI ablation therapy and follow-up. He received RAI therapy (150 mCi) with post therapy scan visualizing a thyroid remnant with star artifact and redemonstrated the sternal mass. A repeat CT scan showed growth in the sternal mass, thyroid bed remnant, and bilateral cervical lymph nodes, and seemingly static pulmonary nodules. The patient opted for further RAI therapy and delayed further surgery.

Ten months later, after receiving another 200 mCi RAI, post-therapy scan showed mild regression in the cephalocaudal axis of the sternal mass lesion from 4.0 cm to 2.6 cm. Follow up CT four months later reconfirmed the sternal mass regression but demonstrated mixed pulmonary nodule findings with some nodules regressing and new ones appearing. The remnant left thyroid tissue and cervical lymphatic metastases appeared to have grown in size compared to the scan one year earlier. The case was thus designated stage T4N1bM1, NRAS mutated FTC.

## 3 Surgical technique

**Fig. 7:**
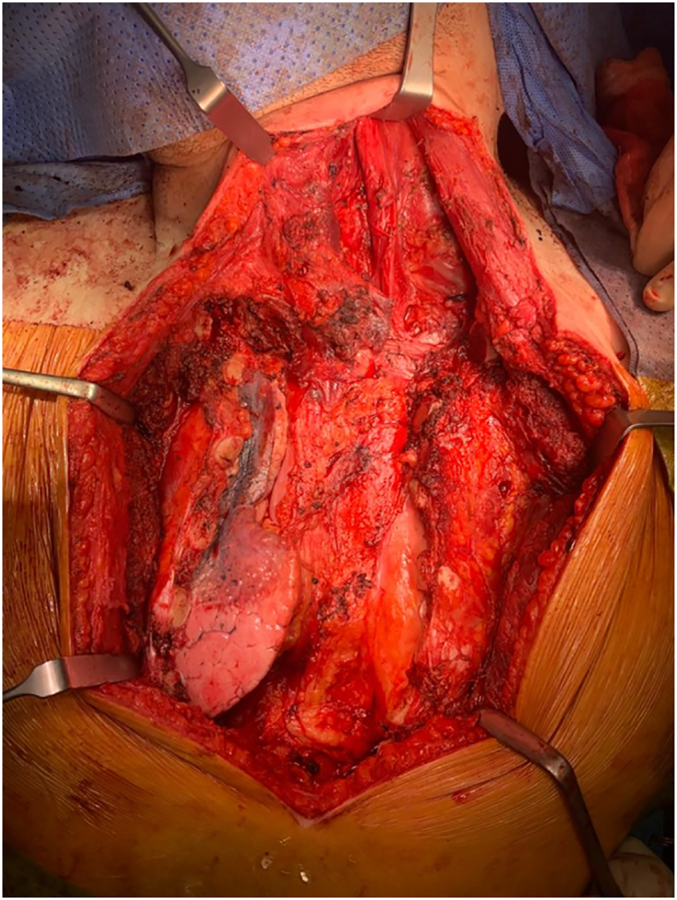
Open chest after excision of sternum.

**Fig. 8:**
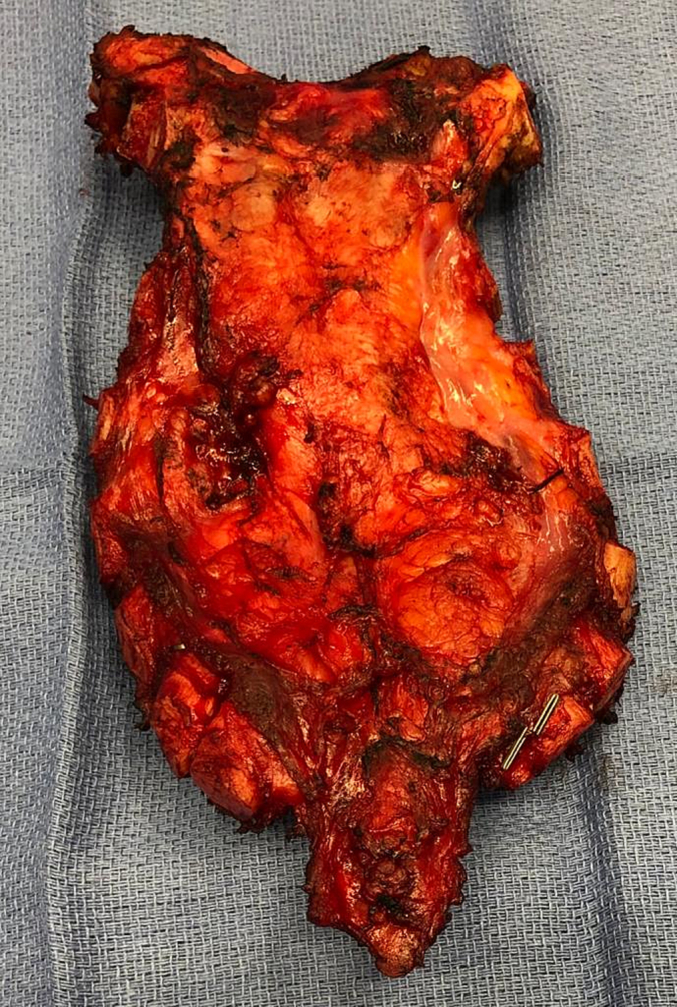
Resected sternum (posterior aspect) with large mass.

Given the above findings, the case was brought to another MDT meeting, which recommended surgical debulking treatment combining General Surgery and Thoracic Surgery to excise the recurrent tumour with left neck dissection and sternectomy with reconstruction (Fig. 7, Fig. 8). The operation was completed successfully with a new sternum prosthesis produced by sandwiching polymethyl methacrylate (PMMA), commonly known as bone cement, between two layers of prolene mesh. Intra-operatively, the tumour was found invading the left internal jugular vein with tumour thrombus forming, hence, the left internal jugular was sacrificed.

The pathology report on the left thyroid nodule specimen was consistent with recurrent FTC (6 × 5 × 2.9 cm) in the left thyroid bed, three tumour deposits in left level VII (1.8 to 4.2 cm) with vascular invasion, 0/5 lymph nodes (LN) in level 5, and a 15 × 6 × 2.5 cm metastatic deposit in the sternum with negative margins. The patient spent four days in the surgical intensive care unit with chest drains. He was later discharged on post-operative day 8, on levothyroxine (LT4) 125 μg daily, with appropriate follow-up instruction provided.

## 4 Post-operative course

A fifth MDT decided that, due to the aggressive nature of the disease, it would be optimal for the patient to receive further high-dose RAI ablation therapy. The patient travelled to Egypt where he received 150 mCi RAI under recombinant human thyrotropin (rhTSH) stimulation (cumulative 650 mCi RAI therapy) (Fig. 1, Section J). His thyroglobulin (TG) levels were significantly elevated (2030 ng/mL). Post-RAI therapy scan showed no uptake, and this increased the suspicion that the malignancy was no longer RAI-avid. PET-CT scan showed persistent disease in left thyroid bed region involving tracheal cartilage, metastatic LNs in subcarinal and right hilar and left preaortic regions, and progression in size and number of bilateral lung nodules, suggestive of recurrence/residual malignancies.

A sixth MDT meeting decided that the metastatic lesions were now most likely RAI-refractory and recommended systemic therapy. The patient was started on Lenvatinib 18 mg daily and planned to continue on LT4 200 μg daily, as he was tolerating well, and was scheduled for regular follow-ups every 6 months (Fig. 1, Section K).

The next follow-up CT showed reduction in the size of the recurrent left thyroid bed lesion, less tracheal encroachment, with redemonstration of stable, cervical and mediastinal LNs, and bilateral lung nodules. Lab values improved (TSH = 0.01 mIU/L, TG = 132.1 ng/mL), and continued to improve, where TG dropped to 106.5 ng/mL. The patient tolerated the systemic therapy well, with adverse effects under control, so it was decided to increase Lenvatinib to 20 mg daily (Fig. 1, Section L).

At the most recent follow-up CT, there was mild reduction in the left paratracheal mass as well as stable sub-centimetric bilateral cervical lymphadenopathy, multiple bilateral lung nodules, and small sub-centimetric mediastinal LNs. Diffuse osteopenia was also noted on CT imaging. Lab values showed TSH = 1.16 mIU/L and TG = 92.8 ng/mL. We further increased his LT4 dose to 250 μg daily.

**Fig. 9:**
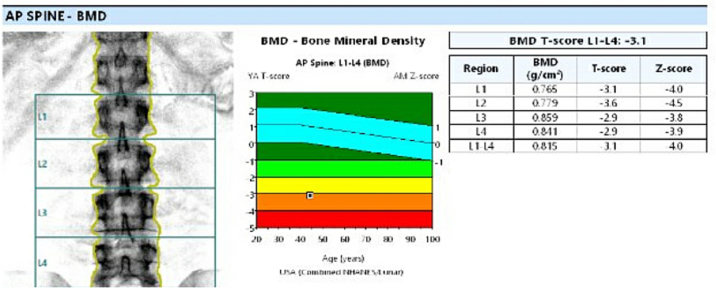
DEXA Scan report showing results consistent with diagnosis of osteoporosis.

As of the most recent update, dual-energy x-ray absorptiometry (DEXA) scan (Fig. 9) showed that bone mineral density was markedly low, an indication of osteoporosis (Fig. 1, Section O). The patient is currently on daily LT4 250 μg and Lenvatinib 20 mg. He is tolerating systemic therapy very well without any reported symptoms (Fig. 1, Section M).

## 5 Discussion

Differentiated thyroid carcinoma are indolent malignancies with favourable prognoses [11]. Bone metastasis (BM) is diagnosed clinically in 4–23 % of DTC patients and is associated with poor prognosis [12]. Secondary malignancy of the sternum is uncommon and mostly encountered among patients with breast carcinoma and less frequently with thyroid carcinoma [13]. We report a 43-year-old Egyptian male with a sternal mass that was found to be malignant thyroid tissue. The primary tumour was then discovered to be bilateral FTC. To our knowledge, this is the second reported case from Africa and the Middle East.

Demographically, our patient was an Egyptian. Whilst a case has been reported from Turkey [14]; within Africa and the Middle East, only one case has been published, namely from Morocco [15]. Our literature review (Table 1) shows that most of the cases reported in the literature were from India [[16], [17], [18]], the Japan, China and Taiwan region [[19], [20], [21], [22], [23]], with isolated cases from USA [[24], [25], [26]], Belgium [27], Germany [28], Greece [29,30], and Malaysia [31]. As for sex, our patient was a male. Table 1 suggests a female predominance across such cases, with a female to male ratio of roughly 2:1. Pertaining to age, the current patient was 43 years old, falling within the age range of 35–75 years reported across the published studies.

**Table 1:** Literature review of cases of thyroid cancer with sternal metastasis.

Study	Age (yr)	Sex	HPr TC	Presentation	TS	Size^a^ (cm)	Diagnosis of TC before SM?	TC subtype	Intervention/repair technique	SC	Other mets	RAI response
Current case	43	M	N	SG SM	Eu	9	Simultaneous	FTC	S; PM sandwiching PMMA	N	Neck, P	Initial +ve, later refractory
Ozaki 1995 [32] Japan	72	F	Y	SG SM	–	4	Y	FTC	S; PMMA fixed with metallic wires	N	Neck, Ribs, V, Fr	–
	51	F	Y	SG painful SM	–	8	Y	PTC	S; MM sandwiching PMMA	N	–	–
Kinoglou 2001 [27] Belgium	62	F	Y	SG SM	–	5	Y	PD FTC	S; MM	N	N	–
Mishra 2001 [18]^b^ India	35	F	N	SG neck & painful SM	Eu	7	Simultaneous	PD TC	S; MM	N	N	–
	43	M	Y	SG SM	–	10	Y	FTC	S; MM	N	P, V, Sacrum	–
Zettinig 2002 [12]^c^ Austria (6 patients)	60 ± 12	–	–	–	–	–	–	–	–	–	–	–
Haraguchi 2004 [19] Japan	69	F	Y	SG painful SM	Eu	14^d^	Y	PTC	S; MM + stainless-steel mesh	N	Neck	+ve
Meyer 2005 [28] Germany	52	F	Y	CT findings of SM	–	–	Y	FTC	S; PM	N	P, Neck	Mixed
Eroglu 2006 [14] Turkey	61	F	Y	SG SM	Hypo	8	Y	FTC	S; Gore-Tex soft-tissue patch + PecMa flaps	–	N	–
Yanagawa 2009 [24] USA	75	F	N	SG neck & SM	Hypo	14^d^	Simultaneous	PD TC	S; PM sandwiching PMMA + PecMa flap	N	P	+ve
Moraitis 2012 [29] Greece	70	F	N	Old neck mass, and slow growing SM	Eu	9	Simultaneous	FTC	S; Gortex dual mesh + PecMa flaps	N	P	+ve
Chen 2013 [23] Taiwan	56	M	N	SG painful SM	–	8	N	FTC	S; PecMa flap	N	V	–
	55	F	N	Long history of goitre (undiagnosed) presenting with SM	–	5.5	Simultaneous	FTC	S; PecMa flap	N	N	–
Ishinaga 2013 [20] Japan	65	F	Y	CT findings of SM	–	2	Y	PTC	S; PMMA/ePTFE composite, PecMa flap	N	N	–
Sabih 2014 [25] USA	66	F	N	SG neck mass	–	8.7	Y	FTC	S; PM + PecMa flap	N	Neck	+ve
Nakayama 2014 [21] Japan (4 patients)^e^	59	–	–	–	–	–	–	–	–	–	–	–
Lan 2017 [22] China	53	F	N	Chronic dull chest pain. CT findings of SM	–	–	Y	FTC	S; Titanium alloy mesh	N	N	–
Sadacharan 2017 [16] India	54	M	Y	Long history of MNG (undiagnosed), presented with SM	Eu	–	Simultaneous	FTC	S; MM	N	P	+ve
Syazni 2017 [31] Malaysia	62	F	N	Long history of MNG (treated), presented with SM	Eu	10	Simultaneous	FTC	S; Titanium mesh, acrylic plate	SSI	P, Skull, V, Pelvis, Fr	+ve
Delliturri 2019 [26] USA	70s	M	Y	SG mass	–	5.8	Y	FTC	S; PMMA, PM + PecMa flap	–	–	–
Pradeep 2020 [17] India	–	F	N	SG neck & sternal mass, dyspnoea	–	6	Simultaneous	PTC	S; Dacron mesh, PMMA, PecMa flap	N	P	–
Id El Haj 2021 [15] Morocco	59	F	N	SG SM	Hypo	7	N	PTC	S; Titanium bars + polymesh dual prosthesis	N	N	–
Paspala 2022 [30] Greece	60	M	Y	SG painful SM	–	4.7	Y	PTC	S; Titanium bars + PM	URTI	N	–

Due to space consideration, only the first author is cited; ePTFE: Expanded polytetrafluoroethylene; Eu: Euthyroid; F: Female; Fr: Femur; FTC: Follicular thyroid carcinoma; HPr TC: History of previous thyroid cancer; Hypo: Hypothyroid; M: Male; mets: Metastasis; MM: Marlex mesh; MNG: Multi-nodular goitre; N: No; P: Pulmonary; PD: Poorly differentiated; PecMa: Pectoralis major; PM: Polypropylene mesh; PMMA: Polymethyl methacrylate; PTC: Papillary thyroid carcinoma; RAI: Radioactive iodine; S: Sternectomy; SC: Surgical Complications; SG: Slow growing; SM: Sternal mass; SSI: Surgical site infection; TC: Thyroid carcinoma; TS: Thyroid status; URTI: Upper respiratory tract infection; V: Vertebrae; yr: Years; Y: Yes; —: Not reported; +ve: Positive.

a Size based on physical exam pre-operatively.

b Metastasis to Sternum, Vertebrae, Sacrum discovered simultaneously.

c Study of 40 patients with bone metastasis from DTC, range: 23–81 years.

d Size measured after excision of sternal mass.

e Study of 41 patients with bone metastases from differentiated thyroid carcinoma.

In terms of previous history of thyroid cancer, the current patient had no history of previous thyroid cancer, although our literature review depicts that many of the published cases had history of previous thyroid cancer [16,18,19,[26], [27], [28],^30^]. This suggests that a metastatic mass to the sternum could, in some cases, be the first indication of an older thyroid cancer that has passed silently undetected. This highlights the need for regular self-examination, and also opportunistic thyroid evaluation as opportunities present themselves when patients utilize the health service for other complaints, in order to aid the early detection of potential thyroid disease before such metastasis occur.

As regards to presentation, our patient initially presented with unusual asthma-like symptoms which initially appeared to be asthmatic. This concurs with a recent case from India, where dyspnoea was feature of the presentation [17], despite that such lung/breathing related symptoms are not common among such cases. Table 1 also depicts that these patients mostly presented with slow growing neck and/or sternal mass, although there have been cases where the mass was rapidly growing [29]. A point is that the description of the rate of tumour growth over the various published reports appears to be subjective, with no clear or consistent timeframe reference as to what constitutes a slow or rapidly growing tumour. More specific reporting in future would be useful to compare rate of tumour growth across different published reports. Pain was not a main complaint of our patient, in agreement with most of the cases in Table 1, although occasionally, some patients reported pain [23,30], or chronic dull chest pain [22].

Regarding size, the sternal mass of our patient measured about 9 cm, slightly towards the larger side, as others observed sternal metastatic masses ranging from a smaller 2 cm [20] to larger 14 cm masses [24]. With regard to laterality of the thyroid malignancy, two reports documented bilateral thyroid nodules [17,20], seven reports described unilateral thyroid malignancy, with two left-sided [26,32] and five right-sided nodules [22,23,25,30,31], whilst three other reports described a multinodular goitre, pointing towards a diffusely enlarged and involved thyroid gland [16,18,29]. Many reports fail to precisely report the side of the thyroid with the malignancy [14,15,18,19,24,27,28]. In future, better reporting will facilitate more meaningful comparisons.

In terms of other metastasis, our case and most patients had some neck metastasis accompanying the sternal metastasis, congruent with other reports [25,28]. One published case reported vertebral metastasis [23], and another had multiple bone deposits in the skull, pelvis, vertebrae, and femur [31]. More commonly, pulmonary metastasis has been widely documented [[16], [17], [18],^24^,^28^,^29^,^31^]. Interestingly, in cases with pulmonary metastasis, these did not seem to cause lung symptoms in most of those patients. This suggests that the infrequently encountered lung symptoms such asthma-like symptoms in the current case or the dyspnoea reported in another [17] appear to be more likely to be caused by compression effects of the sternal mass due to its critical location rather than actual metastatic deposition in the lung/s.

With respect to the subtypes of the thyroid malignancy, the current case was an FTC. Table 1 shows that this type is more commonly encountered among patients with metastatic sternal masses, with a ratio of roughly 2:1 when compared with sternal masses where the primary is PTC [15,17,19,20,30,32]. Even less common is poorly differentiated thyroid carcinoma [18,24]. Our findings are congruent with the literature where bone metastasis is more commonly associated with FTC than with PTC [33].

As for surgery, we undertook prolene mesh sandwiching. Prolene mesh has been widely used with sandwiching or with other procedures [[24], [25], [26],^28^,^30^]. A range of surgical interventions have also been described for removing the sternal mass (Table 1), including Marlex [18,27] or Marlex and stainless-steel mesh [19]. Likewise, Titanium bars have also been used [15,30], or Titanium alloy mesh [22], Titanium mesh and acrylic plate [31] or Gore-Tex soft-tissue patch [14]. Despite that some authors have confirmed the preference to replace the excised sternum with some synthetic substitute [27], some chose to conclude the sternectomy procedure with primary closure and the use of meshes [16,18,19,22,27,28], while others opted for the combine use of a mesh with pectoralis muscle flap [25], or a simpler approach by using only muscle flaps [23]. Future research would benefit from examining the relative effectiveness of such techniques in order to progress the evidence base.

We observed no surgical complications for our patient, in agreement with the literature where most cases had no surgical complications. However, two cases documented complications, the first had surgical site infection [31], and the second had URTI [30]. Notwithstanding there is no clear evidence to suggest that the case of post-operative URTI was a direct result of the disease process or surgical intervention, but rather an incidental outcome.

With respect to management, after excision of the sternal mass, although most case reports mentioned implementing RAI ablation therapy, few described the response, or the changes found on follow up post-therapy scans (Table 1). The few reports that did report such findings, mostly indicated a good response to RAI therapy [16,19,24,25,28,29,31], which concurs with the current patient.

In all cases, after thyroidectomy to excise the primary tumour, thyroxine hormone replacement was initiated, prescribed as LT4 in our case. Prescribing LT4 after thyroidectomy is not only to replace the essential hormone that is no longer endogenously produced, but also to sustain a negative-feedback mechanism on TSH production by the pituitary gland, thought to play a role in suppressing tumour growth [34].

Others have noted that most patients with FTC present in a clinical or biochemical euthyroid state [2]. Not all the reports identified in our literature review reported their patients' thyroid status explicitly in the form of TSH and free T4 levels. However, the clinical picture that was described in each report appeared to suggest that all patients were clinically euthyroid, i.e., not complaining of or presenting with thyroid hormone related symptoms [[14], [15], [16], [17], [18], [19], [20],[22], [23], [24], [25], [26], [27], [28], [29], [30], [31], [32]]. Notwithstanding, three reports observed high TSH levels, which would suggest a hypothyroid state [14,15,24], although the clinical picture provided in these case reports did not indicate any clear-cut hypothyroid symptoms.

Through the clinical course of our patient, biopsy and excised specimens were submitted for gene sequencing, which reported the presence of RAS mutation in Exon 3 of the NRAS gene. The published literature suggests that although the exact role this mutation plays in the outcomes of FTC is not clear, evidence suggests it plays a role in metastasis [35]. None of the case reports in our literature review reported undertaking gene sequencing nor any genetic mutations.

## 6 Conclusion

This case shows that surgical treatment could be a viable therapeutic option for sternal metastasis of FTC. However, there appears no widely agreed upon consensus on the material for reconstruction of the sternum. We used PMMA bone cement. Other options include titanium alloy mesh, biomaterials, and vascularized allografts. Future research is required on the management of FTC with bone metastasis, given its documented poor response to RAI therapy, with surgical excision and reconstruction being an option. Given the prevalence of thyroid cancer, more vigilance is required when assessing patients presenting with respiratory complaints in unusual clinical presentations. Most importantly, regular follow-up and emphasis on continuity of care is vital when discussing a plan of care.

## Provenance and peer review

Not commissioned, externally peer-reviewed.

## Consent

Written informed consent was obtained from the patient for publication of this case report and accompanying images. This is available for the Editor-in-Chief of this journal on request.

## Funding

Nothing to declare.

## Ethical approval

Approved by Medical Research Centre at our institution (MRC-04-23-596).

## Author contribution

**Mohamed S. Al Hassan**: study concept, data interpretation, editing and reviewing the paper. Walid El Ansari: study concept, data interpretation, writing the paper. **Hamza Said Wali**: data interpretation, writing and reviewing the paper. **Ehab Massad**: Reviewing the paper. **Adham Darweesh**: reviewing the paper. **Abdelrahman Abdelaal**: study concept, data interpretation, writing and reviewing the paper. All authors read and approved the final version.

## Guarantor

Prof. Dr. Walid El Ansari.

## Research registration number

Not first in Man.

## Declaration of competing interest

Nothing to declare.
